# The Influence of Rehabilitation Programs on the Mental State and Quality of Life in Patients with Fibromyalgia: A Comparative Cohort Study from Romania

**DOI:** 10.3390/ijerph22101553

**Published:** 2025-10-12

**Authors:** Theodora Florica Borze (Ursu), Annamaria Pallag, Doriana Ioana Ciobanu, Klara Kalman, Anca Paula Ciurba, Ramona Nicoleta Suciu, Mariana Mureșan, Carmen Delia Nistor-Cseppento

**Affiliations:** 1 Doctoral School of Biomedical Sciences, Faculty of Medicine and Pharmacy, University of Oradea, 410073 Oradea, Romania; borze.theodoraflorica@student.uoradea.ro (T.F.B.); mmuresan@uoradea.ro (M.M.); 2 Department of Physical Education, Sport and Physical Therapy, Faculty of Geography, Tourism and Sports, University of Oradea, 410087 Oradea, Romania; dciobanu@uoradea.ro (D.I.C.); kalman.klara@uoradea.ro (K.K.); anca.ciurba@didactic.uoradea.ro (A.P.C.); 3 Department of Pharmacy, Faculty of Medicine and Pharmacy University of Oradea, 410073 Oradea, Romania; 4 Laboratory Loterr, University of Lorraine, 57950 Metz, France; 5 Department of Psycho-Neuroscience and Recovery, Faculty of Medicine and Pharmacy, University of Oradea, 410073 Oradea, Romania; rnsuciu@uoradea.ro (R.N.S.); dcseppento@uoradea.ro (C.D.N.-C.); 6 Department of Preclinical Disciplines, Faculty of Medicine and Pharmacy, University of Oradea, 410073 Oradea, Romania

**Keywords:** fibromyalgia, anxiety, relaxation, physical therapy, hydrokinetotherapy

## Abstract

Fibromyalgia (FM) affects millions of people around the world, causing widespread physical pain, exhaustion, and psychological disorders. Through this study, we aim to observe the effectiveness of two different rehabilitation programs in reducing the impact of FM on daily functioning and psychological factors. Specifically, we compare a complex conservative program that combines physical therapy and hydrokinetotherapy in a hospital setting with a therapy focused on intrinsic relaxation. Methods: This comparative study involved 63 patients aged between 19 and 69 years diagnosed with FM, divided into two groups: the study group (SG, 32 participants) and the control group (CG, 31 participants). Over 90% of participants are female, 30 in the study group and 28 in the control group. SG followed a conservative physiotherapy combined with thermal water therapy, and CG followed a recovery program through intrinsic relaxation. Participants were evaluated on the first and last day of the rehabilitation program using the Revised Fibromyalgia Impact Questionnaire (FIQR) and the Hamilton Anxiety Rating Scale (Ham—A). The rehabilitation program consisted of 10 sessions conducted over a period of two weeks. Results: After the two-week recovery period, the results showed a significant improvement in both FIQR and Ham—A scores in the study group (*p* < 0.001). In the control group, there were no significant changes in FIQR variables (*p* > 0.05), while a significant improvement was observed on the anxiety scale (*p* < 0.001). Conclusions: The combination of hydrokineto-therapy and physical therapy is more effective in improving the overall condition of patients with FM compared to relaxation.

## 1. Introduction

Fibromyalgia (FM) is a complex syndrome characterized by symptoms that affect quality of life [1]. Unlike most rheumatic diseases, fibromyalgia is not considered an inflammatory or autoimmune disease. Biological signs of inflammation in blood tests are generally normal. FM does not cause joint deformities or lead to muscle degeneration. It affects 4.7% of the European population, and approximately 3% of the Romanian population suffers from rheumatic diseases such as arthritis, ankylosing spondylitis, lupus, and others. However, determining the exact prevalence of FM is difficult because many patients are underdiagnosed [2]. Fibromyalgia affects between 0.2% and 6.6% of the world’s population, particularly women between the ages of 30 and 50 [3,4]. It is not a progressive disease that worsens over time, but is associated with decreased social functioning, quality of life and poor mental health [5]. This condition is much more prevalent among women, with prevalence rates varying across countries [6]. Improving the quality of life contributes to a more stable, productive and harmonious life, promoting general well-being. From a medical point of view, this influences the individual’s ability to perform daily work and social functioning through the presence of depression, anxiety, and stress. Therefore, the ability to perform daily roles will be reduced, and, ultimately, patients will develop a different perception of health [7].

The volume of research on pathophysiology is constantly growing. According to some authors, genetic research has revealed an association between FM and conditions such as depressive disorder [8]. Anxiety is a natural reaction of the body to stress or to situations perceived to be threatening [9]. Persistent fear of pain may contribute to the development of psychological problems, including generalized depression and anxiety. Some studies suggest that these conditions may be associated with alterations in monoaminergic neurotransmission, characterized by increased levels of excitatory neurotransmitters such as glutamate and substance P, and decreased serotonin [10]. Possible dopamine dysregulation and alterations in endogenous brain opioids have also been reported [11]. However, these mechanisms should be interpreted with caution and considered more of a hypothesis.

Clinically, women with fibromyalgia often experience stress, anxiety, sleep disturbances, nutritional problems, and body image dissatisfaction [12,13]. In addition, compared with the general population and patients with other specific pain conditions, individuals with fibromyalgia have significantly lower scores on health status assessments using the SF-36 and SF-12 questionnaires [14].

Anxiety treatment includes behavioral therapy, medication, relaxation techniques, physical therapy, and a healthy lifestyle. In long-term recovery, non-pharmacological treatments are prioritized, while medication serves as an adjunct in managing chronic pain. Although it presents a varied clinical picture, chronic pain [15,16] negatively affects both physical and mental functioning [17]. Therefore, treatment is highly complex and multidisciplinary, comprising both physical and mental exercises, and it is carried out in stages, with the first objective being to reduce the negative impact on quality of life [18,19]. This objective can be achieved through intrinsic relaxation, which promotes psycho-muscular inhibition and restores internal well-being. A review of the specialized literature shows that studies on mind–body recovery are limited and require further documentation [20,21]. Online recovery programs help track patients from several perspectives, such as daily environmental stressors, resilience planning and coping styles. At the same time, a home-based intervention can provide long-term benefits, as it can be performed daily without requiring participants to modify their work schedule. All of this aims to verify the effectiveness of treatment, understand the patient’s weaknesses and enable sustained adherence to recovery during a pandemic [22,23,24,25,26].

Hydrokinetotherapy is a globally recommended treatment by doctors for the recovery of musculoskeletal disorders because it combines the effects of water with those of physical exercise [27]. In patients with fibromyalgia, water therapy is indicated due to its analgesic effect and improvement in general conditions [28]. Pain can be relieved by hydrostatic pressure and the effects of temperature on nerve endings that induce muscle relaxation. Chemically, the thermal water in the Băile Felix area is weakly radioactive, containing less than 0.037 microcuries (Ci) per liter and a mineralization of approximately one gram per liter. This water also contains sodium, potassium, calcium, sulfates, magnesium and iron. Its temperature reaches up to 49 degrees Celsius, during recovery using water at 36–37 degrees. The benefits of the oligo-mineral water from Băile Felix have been recognized since 1700. Thermal factors act primarily on the skin, influencing its temperature, vascular volume, and blood circulation, thereby accelerating the recovery process. The chemical component of thermal water benefits the neuro-immuno-endocrine system and exhibits anti-inflammatory, antioxidant, and immunomodulatory activity [29,30]. Thermal baths increase beta-endorphin levels, thereby enhancing their analgesic and antispasmodic effects—an essential objective in the recovery of patients with FM [31]. Aquatic therapy facilitates exercise by reducing water impact, thereby supporting joint mobilization, increasing muscle strength and cardiovascular capacity, and enhancing physical well-being [32].

The objective of this study is to alleviate the symptoms experienced by patients with fibromyalgia that affect their mental state and quality of life. At the end of the study, we expected that patients would be able to better manage stressful situations, facilitated by cortisol reduction through muscle relaxation and physical exercise. At the same time, relaxation techniques help to better focus and calm thoughts, increase motivation and mental energy and improve circadian rhythm. Thus, the patient develops self-confidence and learns to listen to their body and identify the source of physical or emotional discomfort.

In conducting the study, we start from the hypothesis that a multidisciplinary therapy will have the desired effects on patients with FM. We wanted to compare multidisciplinary therapy with intrinsic relaxation therapy because chronic patients need increased adherence to treatment [33]. In this case, home-based recovery could bring long-term benefits. So, we want to see if a single, non-invasive therapy carried out in the comfort of one’s home can also reduce the negative impact of FM in order to offer a cost-free alternative that will visibly improve their quality of life. Thus, by analyzing the results of this study, we will be able to propose to patients a therapy that can be performed daily.

## 2. Materials and Methods

This study observes the effectiveness of two different treatments on the quality of life of patients with FM. The study was conducted in accordance with the Declaration of Helsinki and was approved by the Ethical Committee of the Faculty of Medicine and Pharmacy of Oradea (approval No. 22 of 26 February 2021). All patients were informed about the study and provided written consent for the processing of personal data.

Following the presentation to the rheumatologist, the subjects were informed about the study. Inclusion criteria were: age between 18 and 70 years, a confirmed diagnosis of fibromyalgia, voluntary participation, and absence of contraindications for intervention. Exclusion criteria included failure to attend the assessment, absence from recovery sessions, or the use of medications other than those recorded at baseline. Another rejection criterion was having undergone another type of treatment (such as oxygen therapy, Pilates, and so on) 6 months before or during the study-specific intervention.

Initially, 70 patients diagnosed with fibromyalgia according to the 2016 ACR diagnostic criteria were recruited. [34]. Patients were selected between April 2024 and April 2025. Following application of the exclusion criteria, 7 patients were removed because they engaged in supervised physical activity 3–4 times per week. The 63 selected patients were randomly assigned to two groups, without prior knowledge of symptom severity. Randomization was simple. At the time of presentation to the doctor, each eligible participant was given a number randomly assigned by a computer. Those with even numbers were assigned to the study group (SG) and those with odd numbers to the control group (CG). The SG (*n* = 32) followed a program of physical therapy and thermal water therapy at the Băile Felix Recovery Hospital (Romania).

The study group followed a 20 min program of group aquatic exercises led by a physiotherapist positioned at the poolside. The group rehabilitation program included mobility exercises for the spine, upper limbs and lower limbs. The exercises were performed while standing at a water temperature of 36–37 degrees Celsius, which facilitates movement and reduces gravitational forces. They also had 30 min of individual physical therapy that included warm-up exercises, mobilization exercises for all joints, followed by muscle strengthening exercises and stretching at the end for increased flexibility. Each exercise was performed in four sets of 10 repetitions, synchronized with the breathing rhythm. This program has effects on the cardiovascular and locomotor systems and on mental state.

The CG (*n* = 31) completed 10 home—based recovery sessions, without direct supervision by a physiotherapist. Participants received instructions on how to perform the technique. These sessions consisted of 20 min of relaxation specific to the author Parow, every evening before bed [35]. Parow’s relaxation technique is based on intrinsic relaxation, which ensures mutual inhibition between muscles and psyche. This general relaxation was achieved through a breathing technique. Subjects maintained the position of dorsal decubitus in bed for 20 min, breathing unforced. Inhalation was performed through the nose and exhalation through the mouth, with exhalation lasting twice as long as inspiration. It also involves the integration of an accessible recovery program that can be followed from the comfort of one’s own home. Patients were surveyed online on a daily basis by a physiotherapist from the research team.

In the study, both groups underwent an initial assessment on the first day of recovery. Over the next two weeks, they attended 10 days of treatment sessions. Finally, the last evaluation was conducted on the final day of the study.

### 2.1. Assessments

The two variables analyzed in the study were selected prior to the evaluations, following a review of the specialized literature. Accordingly, patients answered these predefined questions both before and after the recovery sessions. To minimize bias, both the initial and final assessments were conducted by the same assessor.

The negative impact of FM on patients was assessed using the Revised Fibromyalgia Impact Questionnaire (FIQR), a form translated for Romania, validated by ePROVIDE [36]. It targets 3 subfields: function, overall impact, and symptoms. All questions are based on a numerical rating scale of 11 points from 0 to 10, with 10 being the worst. The total FIQR is the sum of the three modified domain scores, where a result ranging from 0 to 42 represents a mild impact of fibromyalgia on quality of life, a moderate impact between 43 and 59, according to the study, a severe impact between 60 and 74, and an extreme impact from 75 to 100.

Anxiety was assessed using the Hamilton Anxiety Scale (Ham-A), the Romanian-translated version [37]. Scala Ham-A is a 14-item semi-structured interview developed by Hamilton in 1959, which generated a number of anxiety-related symptoms and grouped these symptoms into 14 classes. It is sensitive enough to appreciate the change under anxiolytic treatment. The interviewer evaluates the severity of symptoms on a scale of 0 to 4 points at each item, where 0 represents the lack of anxiety, 1—mild anxiety, 2—moderate anxiety, 3—severe anxiety, and 4—disabling anxiety. The odds are summed up in a total score that can be from 0 to 56, with scores in the range of 0–4 indicating a normal score, 5–10 mild anxiety, 11–16 moderate anxiety, and +17 severe, invalidating anxiety.

### 2.2. Statistical Analysis

The statistical processing of the data in the study was carried out using IBM SPSS Statistics for Windows, Version 29.0 (30-day trial version), Armonk, NY, USA: IBM Corp [38]. Qualitative data were presented by a frequency distribution and quantitative data by the mean, median and standard deviation. The student`s t-test was used for continuous variables and the analysis of the association between the categorical variables was made using the cross table, the Fisher test and the test χ^2^ (chi-square). A value of the coefficient of statistical significance *p* < 0.05 was considered significant.

### 2.3. Sample Size and Randomization

The sample size required to detect differences between groups was calculated using the following formula: *n* = (Zα/2 + Zβ)2⋅(2σ2)/d2, where

*n* is the sample size; Zα/2 = 1.96 is the critical value of the standard normal distribution; Zβ = 0.80 is the critical value of the standard normal distribution corresponding to the power of the test (1—β); σ is the population standard deviation, and d is the minimum effect size we want to detect. The minimal sample needed for the study was estimated to be 62 patients. The sample size was calculated using a 95% confidence interval and a 5% margin of error. In our study, 70 patients were enrolled but 63 agreed to participate in the study. Simple randomization was performed at the time of acceptance of participation in the study. Also, randomization was performed before the assessments to avoid bias.

Although the hospital staff were not blinded, they were not involved in the assessments, as all assessments were carried out by the same person. The assessor was blinded, which reduces the risk of measurement bias. Data entry and intervention were not blinded, as therapy data were available to all those involved.

## 3. Results

### 3.1. General Characteristics

In Table 1, it can be seen that there were no statistically significant differences in baseline characteristics, suggesting comparability between groups.

In Table 2, it can be seen that the average age of patients in the two groups shows a difference of approximately 2 years. Ages range from 19 years (minimum) to 69 years (maximum), indicating a wide range of ages within the sample. This difference is not statistically significant, as the *p*-value is much higher than 0.05, suggesting comparability between groups. The standard deviation in both groups suggests moderate variability around the mean. And the standard deviation shows that the ages are fairly distributed around the mean.

Table 3 shows that the majority of participants (60%) fall within the normal weight range. However, 36.6% of participants fall into the overweight or obese categories, indicating a substantial proportion of body mass index (BMI) values above the normal range, while 3.3% are underweight. In the CG, the majority of participants (60%) were in the normal weight category. However, 36.7% were classified as overweight, and 3.3% underweight. The mean BMI of participants was 23.63, placing the majority within the normal weight range. However, the wide range of values (from underweight to overweight) suggests heterogeneity in body weight within the sample. The *p* value for body mass index is greater than the significance threshold of 0.05.

### 3.2. Results of the FIQR Score

The analysis of the data in Table 4 indicates statistically significant differences (*p* < 0.05). The between-group comparison showed significant differences at baseline, but not at the final assessment (*p* = 0.08). The largest effect was observed in the total FIQR score, with a mean difference of 16.68 points and a final value of 49.83. The Minimum Clinically Important Difference (MCID) is generally accepted as 14–27% of the total score, corresponding to 8.1–12.8 points. In the study group, the mean improvement of 16.7 points exceeded the MCID threshold, indicating a clinically meaningful effect. Individual-level analysis revealed that 22 (68.75%) patients achieved reductions of 13.5–26.17 points, exceeding the MCID, while 6 patients had clinically meaningful decreases of 8.83–12.66 points. Four patients showed reductions of 6.8–8.0 points, which fall below the MCID threshold. In contrast, the control group showed a mean decrease in FIQR score from 56.59 to 54.81, which remained below the MCID. At the individual level, no patient demonstrated an improvement greater than 8 points.

**Figure 1:**
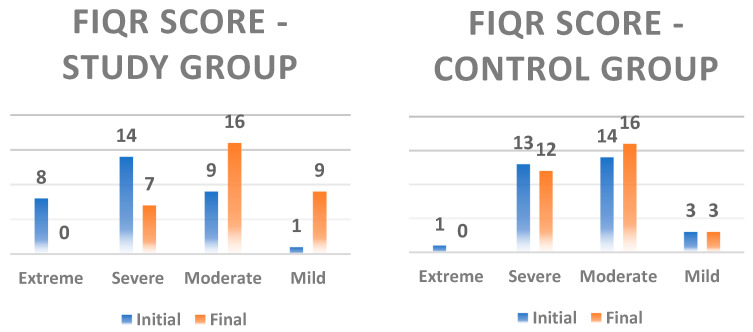
Evolution of FIQR scores from initial to final evaluation in the two groups. The quantitative details represent the number of patients in each level of the scale, where a FIQR score between 0 and 42 points represents a mild level of anxiety, between 43 and 59, a moderate level, between 60 and 74, a severe level of anxiety, and above 75, an extreme level of anxiety.

Figure 1 also shows that extreme scores were reduced in both groups, with a more pronounced reduction observed in the study group.

#### Comparison of Average FIQR Scores by Dimensions at the Initial and Final Moment

The analysis of the data between the groups in Table 5 indicates that there were significant differences between the initial average values on all three evaluated dimensions. In terms of the second assessment, differences are found only in symptoms, without differences in function and overall impact, suggesting that the overall impact of symptoms remained constant in both groups. These differences suggest that the study group might have higher symptom severity or impaired functioning compared to the control group at the start.

All areas measured in the study group (total FIQR, function, overall impact, symptoms) showed significant improvements between initial and final measurements (*p* < 0.001). The effect size of 0.90 points on the FIQR suggests a general improvement in health status by reducing symptoms, improving functionality, and reducing the general impact of FM. In the control group, all measured FIQR variables show changes, but not statistically significant, *p* > 0.05, except for the total function domain, which shows a constant state between the two evaluations with a moderate effect size.

### 3.3. Assessment of Anxiety

The effect size of 0.87 points on the Hamilton scale in the study group (24.57 ± 7.51 initial versus 16.37 ± 5.05 final) indicates a significant improvement in the patient’s condition. In the control group (19.47 ± 6.01 versus 15.07 ± 4.87), the mean difference of 4.40 is statistically significant, indicating an improvement in health status as measured by the Hamilton scale, with an effect size of 0.89 points.

**Figure 2:**
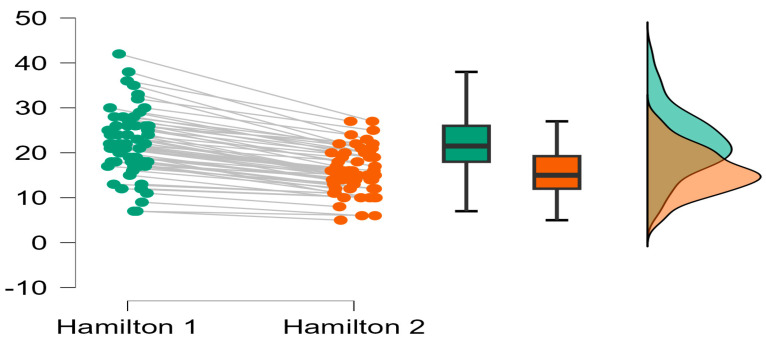
The evolution of the Hamilton score at the cohort level. The green color represents the distribution of the results of the evaluation of the Ham-A variable at the initial moment and the orange color represents the distribution of the results of the evaluation of the Ham-A variable at the final moment.

At the cohort level, the Hamilton score decreases significantly (*p* < 0.05) from the first evaluation to the second evaluation. Figure 2 suggests the improvement of the Hamilton score obtained in the two evaluations.

In Table 6, it can be seen that at the final assessment, the difference between the groups was not statistically significant. From the point of view of MCID, both groups showed clinically significant improvements, exceeding the MCID threshold. The accepted MCID value for this variable is 2–3 points, or a 20% reduction from the initial score. In the study group, the mean score decreased from 24.57 points to 16.37, representing a reduction of 8.2 points (approximately 33%). All patients showed a decrease of at least 2 points: 3 patients had a reduction of 2 points, while the remaining 29 (90.62%) achieved reductions between 5 and 14 points, exceeding the MCID.

In the control group, the mean score decreased from 19.47 to 15.07, representing a reduction of 4.4 points (approximately 22%), which is also clinically significant. One patient had results below the MCID threshold, 6 patients had reductions between 2 and 3 points, and the remaining 24 (77.41) exceeded the MCID, with a maximum reduction of 7 points.

### 3.4. Correlation of Function with Anxiety

**Figure 3:**
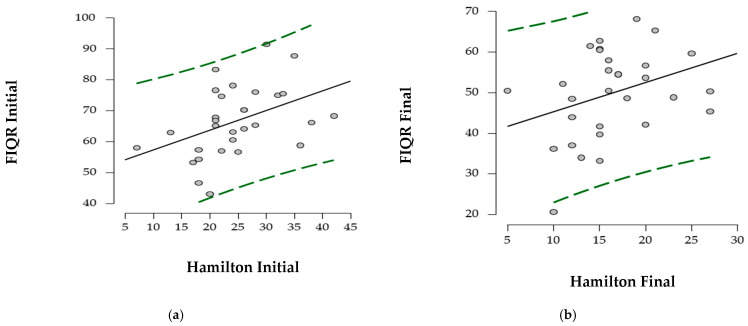
Correlation of function with anxiety. (**a**) initial; (**b**) final. The points suggest the distribution of scores at the initial and final times of the two assessments and the correlation between them.

The relationship between the FIQR score and the initial and final Hamilton score was evaluated in Figure 3. The data analysis suggests a moderate association of the initial and final values between the two scores (r = 0.424 and r = 0.333, respectively).

## 4. Discussion

Considering the objective of the study, the results show that the intervention applied to the study group led to significant improvements in FIQR scores, function and anxiety. This is also observed through MCID, which, according to the literature, indicates clinically significant improvements [36,39,40,41]. Referring to the hypothesis, in the control group, the reduction in anxiety helps patients better manage the negative effects of fibromyalgia, leaving aside daily anxiety and its consequences. But regarding function, the hypothesis was not confirmed, since no clinically significant improvements were recorded.

These results are consistent with the existing literature: the beneficial effects of thermal water have been known since antiquity, by reducing forces on joints and facilitating physical exercise [42]. Hydrokinetotherapy reduces physical stress and muscle tension, favoring oxygenation of the whole body. This mechanism may explain the significant decrease in FIQR and Hamilton scores in the study group. Previous studies confirm the positive effects of balneotherapy on patients with fibromyalgia, including reduction in physical symptoms, improvement of anxiety and depression, and increased functionality [43,44,45].

The literature suggests that mind–body therapies are effective in improving quality of life and managing psychological distress associated with fibromyalgia [46,47,48,49]. Our results confirm this observation.

The moderate correlation between FIQR and Hamilton scores suggests that improved physical function is associated with reduced anxiety, and interventions combining exercise with aquatic therapy may provide greater benefits than passive measures. On the other hand, the effects of relaxation therapy on quality of life are small but are beneficial for anxiety. The reduction in anxiety observed in the control group may be explained by the reduced impact of fibromyalgia on daily activities [50]. Hou Q et al. argue that the duration and severity of the disease influence the response to treatment, with chronic patients having poorer outcomes due to physical and psychological stress [51]. Our results indicate that multidisciplinary interventions may be an effective option even for patients with moderate forms of fibromyalgia.

All patients in the study used anti-inflammatory drugs both before and during the trial. They had not engaged in regular physical activity previously and had only attempted drug therapies to alleviate symptoms. The similarity of prior treatments and the homogeneity of group characteristics suggest that observed improvements can be largely attributed to the intervention, although individual motivation and response to medication should also be considered. Despite the study group presenting with more severe FIQR and HAM-A scores, group allocation was randomized prior to evaluations.

The control group was recommended to perform the 10 recovery sessions within the Hospital.

### Strengths and Limitations of the Study

This study has several strengths. It is the first study in Romania to evaluate and compare the effectiveness of hydrokinetotherapy in patients with fibromyalgia, providing new and clinically relevant data, local evidence being limited. The intervention was standardized and validated instruments (FIQR and Hamilton scale) were used to assess functional and psychological outcomes. All assessments were performed by a blinded evaluator, which reduces the risk of measurement bias.

However, there were some limitations. First, the study period was relatively short, due to national regulations requiring a minimum of 10 free sessions every 6 months. This limited the assessment of long-term effects, although a 6-month follow-up is planned. Second, the sample size was modest, reflecting both the low prevalence and diagnostic difficulties of fibromyalgia, thus limiting the detection of smaller differences between groups. Another limitation concerns the comparability of the groups. At baseline, the study group had higher FIQR and Hamilton scores, suggesting more severe symptoms compared with the control group. These differences at baseline could have influenced the improvements observed in the study group. Regarding blinding, although the assessor was blinded, the patients and therapists were not, due to the nature of the intervention. This lack of treatment blinding could have introduced performance bias. In addition, the control group underwent home-based recovery sessions, which could have negatively affected adherence and perceived support compared to supervised therapy in the hospital, potentially reducing the effectiveness of the intervention in this group.

Despite these limitations, from a clinical perspective, the study offers important future prospects. The results demonstrate that thermal water hydrokinetotherapy can produce statistically and clinically significant improvements in function and anxiety in patients with fibromyalgia. Future research should investigate the impact of treatment adherence after 6 months of relaxation therapy, include multicenter cohorts, and extend the follow-up period.

## 5. Conclusions

Thermal water in combination with physical therapy provides significant improvements in the negative impact of fibromyalgia and reduces psychological symptoms, thus proving the effectiveness of the intervention. At the same time, exercises performed in thermal water at 36 degrees reduce pain and promote blood circulation and muscle relaxation, leading to increased self-esteem. The relaxation therapy has lower effects on the analyzed variables, reducing only the level of anxiety. Thus, although the effectiveness of relaxation on psychological parameters can be demonstrated, for functional parameters, it is also necessary to integrate a physiotherapy program. Therefore, combining the two therapies would maximize therapeutic effects and could be recommended in the management of patients with FM.

## Figures and Tables

**Table 1:** Baseline patient demographic characteristics.

Characteristics	Study Group	Control Group	*p*-Value
Female, N (%)	30 (93.75)	28 (90.32)	0.810
Male, N (%)	2 (6.25)	3 (9.67)	0.672
Use of medication NSAID	32 (100)	31 (100)	-

NSAID = nonsteroidal anti-inflammatory drugs; *p* = statistical significance coefficient.

**Table 2:** Data on the age of the subjects.

Age	Group	N	Min.	Max.	Mean	SD	Std. Error Mean	t	*p*
	SG	32	19	68	46.95	13.16	2.403	0.705	0.485
	CG	31	25	69	44.74	11.31	2.066		

SD—standard deviation; N—number of patients; *p* = statistical significance coefficient; Min. Age—minimum age; Max.—maximum.

**Table 3:** Data on participants’ BMI and statistical comparison.

BMI (kg/m^2^)	Group	N	Min.	Max.	Mean	SD	t	*p*
	SG	32	17.69	36.33	24.50	4.41	0.801	0.421
	CG	31	17.30	29.59	23.63	3.06		

BMI—body mass index; SD—standard deviation; N—number of patients; *p* = statistical significance coefficient; Min.—minimum BMI; Max.—maximum BMI in the group.

**Table 4:** Evolution and comparison of FIQR total scores between groups at the initial and final moments.

Parameter	Group	Mean	SD	t	Mean-D	*p*-Value
FIQR it	CG	56.569	11.466	3.22	9.510	0.002
	SG	66.507	11.258			
FIQR ft	CG	54.819	10.786	−1.78	−4.989	0.08
	SG	49.830	10.894			

FIQR It—the results of the initial evaluation of the FIQR variable; FIQR ft—the results of the final evaluation of the FIQR variable; CG—control group; SG—study group; SD—standard deviation; *p* = statistical significance coefficient; Mean-D—mean difference; it—initial test; ft- final test.

**Table 5:** Comparison of average FIQR scores by dimensions at the initial and final moments.

Parameter	Group	Mean	SD	Coefficient of Variation	t	Mean-D	*p*-Value
FIQR i function	CG	16.230	3.776	0.233	2.32	2.544	0.024
	SG	18.774	4.663	0.248			
FIQR f function	CG	16.230	3.776	0.233	−3.00	−3.133	0.004
	SG	13.097	4.282	0.327			
FIQR i overall impact	CG	13.167	3.239	0.246	2.65	2.167	0.010
	SG	15.333	3.089	0.201			
FIQR f overall impact	CG	12.733	3.005	0.236	−0.46	−0.367	0.647
	SG	12.367	3.157	0.255			
FIQR i symptom	CG	27.200	5.255	0.193	3.33	5.160	0.001
	SG	32.360	6.648	0.205			
FIQR f symptom	CG	25.900	4.779	0.184	−0.82	−1.100	0.412
	SG	24.800	5.497	0.222			

FIQR i function—the results of the initial assessment of the dimension relating to the impairment of function; FIQR f function—the results of the final assessment of the dimension relating to the impairment of function; FIQR i overall impact—the results of the initial assessment of the overall impact; FIQR f overall impact—the results of the final assessment of the overall impact; FIQR i symptom—the results of the initial assessment of the symptom dimension; FIQR f symptom—the results of the final assessment of the symptom dimension; CG—control group; SG—study group; SD—standard deviation; Mean-D—mean difference; *p*—statistical significance coefficient;.

**Table 6:** Comparison of the Hamilton score at the initial and final moments.

Parameter	Group	Mean	SD	t	Mean—D	*p*-Value
Hamilton initial	CG	19.467	6.010	2.90	5.100	0.005
	SG	24.567	7.514			
Hamilton final	CG	15.067	4.877	1.01	1.300	0.315
	SG	16.367	5.055			

CG—control group; SG—study group; SD—standard deviation; Mean-D—mean difference; *p* = statistical significance coefficient.

## Data Availability

The original contributions presented in this study are included in the article.

## Abbreviations

The following abbreviations are used in this manuscript:

FM	Fibromyalgia
FIQR	The Revised Fibromyalgia Impact Questionnaire of FM
Ham—A	Hamilton Anxiety Scale
SG	Study Group
CG	Control Group
